# No effect of short term exposure to gambling like reward systems on post game risk taking

**DOI:** 10.1038/s41598-022-21222-3

**Published:** 2022-10-06

**Authors:** Nicholas J. D’Amico, Aaron Drummond, Kristy de Salas, Ian Lewis, Callan Waugh, Breanna Bannister, James D. Sauer

**Affiliations:** 1 School of Psychological Sciences, University of Tasmania, Hobart, Australia; 2 School of Psychology, Massey University, Palmerston North, New Zealand

**Keywords:** Psychology, Human behaviour

## Abstract

Is engaging with gambling-like video game rewards a risk factor for future gambling? Despite speculation, there are no direct experimental tests of this “gateway hypothesis”. We test a mechanism that might support this pathway: the effects of engaging with gambling-like reward mechanisms on risk-taking. We tested the hypothesis that players exposed to gambling-like rewards (i.e., randomised rewards delivered via a loot box) would show increased risk-taking compared to players in fixed and no reward control conditions. 153 participants (*M*_*age*_ = 25) completed twenty minutes of gameplay—including exposure to one of the three reward conditions—before completing a gamified, online version of the Balloon Analogue Risk Task (BART). Self-reports of gambling and loot box engagement were collected via the Problem Gambling Severity Index, and Risky Loot-Box Index. Bayesian t-tests comparing BART scores across reward conditions provided moderate to strong evidence for a null effect of condition on risk-taking (BF = 4.05–10.64). Null effects were not moderated by players’ problem gambling symptomatology. A Spearman correlation between past loot box engagement and self-reported gambling severity (*r*_*s*_ = 0.35) aligned with existing literature. Our data speak against a “gateway” hypothesis, but add support to the notion that problem gambling symptoms might make players vulnerable to overspending on loot boxes.

In the US and Australia, at least two-thirds of the population play video games^1,2^ and the global gaming market is predicted to reach a value of $314 billion (USD) by 2026^3^. The exponential growth of video gaming has prompted an increase in research into the positive and negative impacts of the activity. Increasingly, research evidence suggests that video game engagement can reach problematic and addictive levels, causing psychological distress to individuals^4,5^.

As the gaming industry grows, and the number of titles competing for gamers’ attention increases, innovations are designed to capture player attention, maintain engagement, and generate income^6,7^. Some researchers attribute the growth of video gaming and gaming-related revenue to innovations in reward design and monetisation strategies, which now include the ability to purchase randomised in-game items (i.e., loot boxes) with cash or virtual currency. These monetisation mechanisms have generated media controversy^8,9^, with some scientists regarding the monetisation of rewards as “predatory” to consumers^10,11^. Loot boxes, in particular, have generated considerable public and academic interest; being likened to gambling due to similarities in both aesthetics, and in the shared psychological mechanisms upon which the two activities rely^6,12,13^. Some researchers and policy makers have speculated that, given these similarities, loot boxes might serve as a “gateway” to future gambling^14,15^. To some, this seems intuitive. To others, unlikely. A critical question, though, is through what mechanism might exposure to gambling-like in-game rewards lead to future gambling? We tested one causal pathway through which engagement with loot boxes *might* increase the risk of the future gambling: via increased risk-taking. Importantly, it was not our intent to test all potential mechanisms which may lead to future gambling behaviour. Rather, we sought to determine whether engagement with the randomisation elements of loot boxes might increase risk-taking behaviour specifically.

## Loot boxes

A loot box is a digital container of rewards within a video game, often purchasable with real money. The different rewards in a loot box are acquired ostensibly at random, with rarer (and more desirable) rewards awarded less frequently than common (and less desirable) ones. Rewards vary from game to game, but often offer a competitive in-game advantage (i.e., unlocking a character or item;^6,16^). Other rewards exclusively offer cosmetic changes to gameplay (e.g., altering the appearance of in-game characters or items), but can nonetheless be desirable to players based on their scarcity or esteem in the gaming community^17^. Loot boxes are increasingly prominent on mobile, console, and PC game platforms^18^. Additionally, for products with an age rating of 7 years and older, almost a billion installations of games containing loot boxes were recorded in U.S. app-stores. Further, an analysis of the most-played games on the PC platform *Steam* from 2010 to 2019 observed an increase in exposure to loot boxes from 5.3% of players in 2010, to 71.2% by 2019^18^. Thus, loot box exposure is increasing across age groups and—given their ubiquity across platforms—across broader gaming demographics. In response to their ubiquity, research has emerged concerning the way loot boxes function as part of the broader gameplay experience, and how they influence player behaviour.

Although many loot boxes sell for less than $3 USD individually, they generate billions of dollars in aggregated post-sale revenue for the gaming industry^6,7,10,19^. Items won from some loot boxes can be on-sold in online marketplaces, and sale prices can far exceed the cost of purchase, indicating the value some rewards have for gamers^20^. However, many items from loot boxes have a resale value lower than the loot box purchase price, meaning that players incur a financial loss when on-selling items won from loot boxes^7^. Due to the random nature of acquiring rewards through loot boxes, players are more likely to receive common items than rarer ones after purchase. This hierarchy of reward-likelihood creates a system of value and rarity for in-game items that does not exist for direct purchase microtransactions, and allows for the delivery of valued outcomes on intermittent reinforcement schedules^21–23^.

### The psychology of loot boxes

The design of many loot box systems appears psychologically similar to traditional forms of gambling, and this is reflected in trends of player behaviour with loot boxes^13,16,24,25^. Drummond and Sauer^6^ utilised Griffiths^26^ framework to show that most loot boxes in popular games meet key psychological criteria for gambling. These criteria include (i) the exchange of money on (ii) an unknown future event with (iii) an outcome determined at least partly by chance, (iv) the ability to avoid losses by opting out, and (v) the ability to “cash out” winnings. Further, the randomisation element central to loot boxes allows for variable ratio reinforcement; a feedback mechanism that promotes behaviour acquisition and repetition, and attenuates behavioural extinction^6,27^. In the context of loot boxes, variable ratio reinforcement would be expected to encourage repeated and frequent purchasing behaviour, even if desired outcomes are delivered with decreasing frequency. These reinforcement features are central to operant conditioning and underpin many traditional gambling activities.

Both conventional gambling and loot box purchasing require a wager on an unknown result, and a financial risk is inherent to the activity. Neither a win nor loss is guaranteed, outcomes are largely determined by chance, and “wins” are distributed intermittently (and sometimes at random, depending on the nature of the specific gambling activity). In this way, virtually every gambling activity, and every loot box purchase both relies on and feeds into operant conditioning and variable reinforcement schedules^6,28^. Given the structural and psychological similarities between loot boxes and conventional forms of gambling, researchers and members of the public have wondered whether engaging with loot boxes may have negative outcomes for gamers. In addition to concerns raised that loot boxes may encourage, at least for some gamers, excessive game time or spending^24^, there has been some speculation that engaging with loot boxes may serve as a “gateway” to future gambling. Although there is a robust positive association between problem gambling symptomatology and loot box spending, there have been no direct experimental tests of this gateway hypothesis and no explicit consideration of the causal pathways through which loot box engagement might contribute to an increase in future gambling. One possibility is that repeated exposure to risk—via engagement with loot boxes—might affect cognitions associated with risk assessment and willingness to engage in future risky behaviour, and these effects might translate into an increased predisposition toward gambling.

### Risk-taking: risk and reward

Risk-taking—voluntary action performed under uncertainty, which carries some possibility for negative consequences^29,30^—is core component of both gambling and loot box purchasing. The development of risk-taking and risk assessment is well documented, increasing from childhood to young adulthood, with a peak and decrease in later adulthood^31^. Although not all risk-taking is problematic, some risk behaviours can be negative (i.e., crime, drug use; violence and aggression;^32,33,34^. However, higher risk-taking in youth is associated with addiction vulnerability later in life^29,31,35^. Thus, adolescent loot box purchasing may have implications for future risk-taking behaviour^36,37^, as risk is an inherent part of loot box engagement and loot box use is considered by some to be a form of risk behaviour^38,39^.

Risk-taking is influenced by the reward outcome of a decision, with some individuals displaying higher sensitivity to reward than others. The motivation to take a risk can be understood through two mechanisms: behavioural *activation* and *inhibition*. Behavioural activation refers to excitation in the presence of a reward, while inhibition governs the interruption of behaviour^40^. Individuals who are risk-averse may be better at inhibiting their thoughts and emotions, while a risk-prone person might have a lower capacity to inhibit their behaviour^41,42^. Rutherford and colleagues^43^ found that youth who engaged in risk behaviours such as underage gambling displayed an imbalance between reward activation and inhibition, resulting in high scores on risk-taking measures.

### Risk and future gambling

The link between gambling and risk is well-established: Gambling inherently involves risk-taking^44–46^, and perceptions of risk play a substantial role in the production and maintenance of gambling behaviour^47^. Armstrong et al.^48^ argued that simulated gambling games may increase risk-taking behaviour due to the cognitive distortions that result from repeated exposure to risk-free simulations, and suggested that engaging with such gambling simulations creates a *gamified illusion* that results in misconceptions about the risks (i.e., potential financial losses, addiction) associated with actual gambling. Such cognitive distortions may also contribute to the formation of habitual behaviours (e.g., chasing losses;^49^). Armstrong et al.^48^ found that youth who engaged with virtual currency and simulated gambling were more vulnerable to later financial and problematic gambling. Thus, concerns have been raised about the accessibility of loot box mechanisms to underage gamers^50^, with studies demonstrating that many popular game titles available to children include loot boxes that meet the psychological criteria for gambling^6^, and that nearly half of young gamers having already engaged with loot boxes^51^. Here, we sought to test the mechanism proposed by Armstrong with regard to loot boxes: that players who are exposed to the randomised elements of loot boxes might subsequently engage in more risk-taking behaviour than players who are not, on one measure of risk-taking behaviour (the Balloon Analogue Risk Taking Scale). Importantly, as our design only tests this specific mechanism, our results do not speak to the presence or absence of other, alternative mechanisms, which may contribute to increased gambling behaviour.

### Loot boxes as a gateway to gambling

Given the established similarities between loot boxes and many conventional forms of gambling, and the relatively low cost (and financial risk) of any individual loot box purchase, loot boxes may be considered examples of relatively low-risk gambling simulations in gaming contexts. Thus, loot boxes may distort perceptions of risk in a manner similar to simulated gambling activity^38^. This distortion may contribute to a *gateway* effect^10,52^ whereby engagement with loot boxes begins a sequence of behaviour that may increase player risk-taking, and act as a precursor to future, potentially problematic, gambling.

Moreover, engaging with loot boxes may act to condition players to further spending, increased risk-taking, and longer play time. As Zendle^53^ notes: “intermittent wins that characterise loot boxes may result in a process of conditioning in which loot box spenders learn to associate gambling-like experiences with excitement” (p. 3). The excitation associated with this conditioning process can be seen in research comparing player brain activity during a loot box opening to activity during gambling tasks. For example, Larche et al.^17^ found that winning a reward from a loot box activated the same neurobiological reward responses as monetary wins from a slot machine, with rarer items in loot boxes eliciting stronger responses.

A consistent positive relationship between loot box purchasing and problem gambling symptoms is established in the literature^16,54–57^. Although this work does not imply a causal relationship between loot box spending and the development of problem gambling symptomatology—and may simply indicate that these mechanisms are disproportionately enticing to those at risk of problem gambling—concerns have been expressed that loot box engagement may have a causal influence on maladaptive behaviours, such as future gambling^9,14,38^. The factors underlying a directional influence between loot box use and risk-taking necessary to support a *gateway hypothesis* have not been established^10^. Regardless of our beliefs about the likelihood of a gateway mechanism in this context, the issue merits investigation because there are some plausible (though not necessarily probable) theoretical pathways for the relationship, and because policy makers are already making claims about the possibility of such a gateway^14^. Thus, some empirical investigation of the relationship is required.

Specifically, the temporal order of the links between loot boxes, gambling, and risk behaviour is yet to be understood. However, Zendle^53^ offers several speculative explanations for such a relationship. First, and by design, loot boxes may act as a gateway to gambling. Second, pre-existing gambling behaviours, tendencies, or predispositions may drive higher loot box purchasing upon engagement. Third, individuals who have access to loot boxes may also be in digital proximity to online gambling products and be more likely to access them, resulting in a co-occurrence of loot box purchasing and gambling. This area of research is in its infancy, as most studies have relied on self-reports of loot box spending, behaviour frequencies, and problematic gambling tendencies. Thus, there is limited research on the immediate effect of loot box engagement on player *behaviour*.

### The present study

In this pre-registered study, we took a first step to address this gap and tested one mechanism that might support a gateway hypothesis; comparing individuals who interact with in-game loot boxes with those who do not, on an established behavioural measure of risk-taking. Risk *behaviour* measures are designed to assess actual risk behaviours (i.e., the “revealed-preferences” approach^58^). Behavioural measures are often designed to capture or demonstrate the cognitive processes underlying risk behaviours^58^. We therefore used a common behavioural risk measure to assess the influence of engaging with gambling-like in-game rewards on player risk-taking: The Balloon Analogue Risk Task (BART;^59,60^).

We recruited participants to play a bespoke videogame (modelled on popular match 3 style games, to maximise participants’ ability to “pick up and play”) in one of three reward conditions. Some participants were exposed to a reward mechanism which allowed them to purchase *randomised rewards*—effectively “power-ups” that helped players accomplish in-game tasks—with currency earned in-game (Loot Box Condition); some participants were exposed to a reward mechanism which allowed them to purchase *visible or known rewards* with currency earned in-game (Fixed Reward condition designed to be akin to a standard microtransaction for in-game content); and other participants played the game without any reward mechanism (No Reward, control condition). After gameplay, participants completed the Balloon Analogue Risk Taking (BART) task, followed by the Problem Gambling Severity Index (PGSI) and Risky Loot Box Inventory (RLI). Including both a randomised rewards (Loot Box) and fixed reward condition was a key experimental manipulation, allowing us to separate the effects of engaging with gambling-like (i.e., randomised) rewards that involve some element of risk (i.e., where the outcome of a transaction might be more or less desirable), from the effects of in-game spending more generally, on behavioural risk-taking.

Given the inherent role of risk-taking in gambling, and the structural similarities between loot boxes and gambling activities: we tested the hypothesis that engaging with loot boxes in-game—as a gambling-like reward mechanism—would be associated with *increased* post-game risk-taking such that:

***H1***: Participants in the Loot Box (random reward) condition would have higher scores on the BART than participants in the No Reward (control) condition.

***H2***: Participants in the Loot Box condition would have higher scores on the BART than participants in the Fixed Reward (non-randomised) condition.

Due to the exploratory nature of this design, we also tested for differences in risk-taking between the No Reward and Fixed Reward condition (***H3***).

Given the interrelationship between risk and gambling, previous gambling experience may influence the relationship between loot box engagement and risk-taking. Thus, we tested if differences in BART scores across the three reward conditions were moderated by participants’ scores on the PGSI such that:

***H4***: Participants with higher PGSI scores would show greater differences in BART scores from the Loot Box to No Reward conditions than participants with lower PGSI scores.

***H5***: Participants with higher PGSI scores would show greater differences in BART scores between the Loot Box to Fixed Reward conditions than participants with lower PGSI scores.

The RLI is a relatively new measure of loot box engagement^38^. Thus, we had the opportunity to test if BART scores were correlated with participants’ scores on the RLI (***H6***) and as a replication of the original study, we tested if RLI scores were correlated with participant PGSI scores in our sample (***H7***).

## Method

### Preregistration

To facilitate transparency, reproducibility, and to demonstrate good research practice^61^, this study—including all hypotheses, Bayesian and Frequentist analyses, transformations, and exclusions—was preregistered on the OpenScience framework (https://osf.io/f4wgh).

### Participants and design

A priori power calculations were conducted in G*Power for a one-tailed independent samples t-test comparing two means and suggested that 51 participants per condition (N = 153) would yield 0.8 power to detect a moderate (*d* = 0.5) difference between any two conditions. Despite our intention to run Bayesian t-tests for our key comparisons, we used this calculation based on Frequentist analyses to guide recruitment cut-offs.

### Data screening

A total of 166 participants completed the experiment between May and September 2021 (Data collection for this study began in 2020 but was interrupted by the COVID19 pandemic, and only 26 participants were tested. We began data collection anew, and collected a full sample of 153 participants, in 2021. However, in the interest of full transparency, we report analyses including the data collected in 2020 in the supplemental materials (SI Tables 1–4). The patterns of results do not change). 13 participants were removed due to incomplete datasets resulting from software issues, leaving 153 participants for analysis. Preregistered exclusion criteria included participants who failed to respond to at least 75% of the PGSI questions, and this did not apply to any of our remaining data.

One hundred and fifty-three participants (91 males, 59 females, 3 other), aged 18 to 53 years (*M* = 25, *SD* = 6) were assigned to one of the three experimental conditions in our between-groups design, with 51 participants in each. Participants were recruited from a variety of sources including the undergraduate student body and social media platforms. All participants entered a prize draw for one of six $50 gift vouchers for completing the experiment.

Across gaming platforms (mobile/tablet, console, and PC), 64 participants reported not gaming at all, and the average reported time spent gaming per day was approx. 2 h and 20 min, with a standard deviation of approx. 4 h. Fifty-three percent of participants reported having previously purchased a loot box.

### Ethics approval

Ethics approval was granted for a minimal risk application (H0021748) by the Tasmania Social Sciences Human Research Ethics Committee at the corresponding author’s institution. The research was performed in accordance with all relevant guidelines/regulations and in accordance with the Declaration of Helsinki. Participation was anonymous and voluntary, and informed consent was obtained from all participants prior to their participation in the study.

### Materials

#### Our game

The Match-3 game used in this research was designed to emulate a popular Match-3 game, which is well known to the general population and accessible (i.e., easy to play) for participants with no prior gaming experience (see supplemental materials, SI Fig. 1). The game requires participants to match coloured candies in rows or columns of three, with each level requiring candy-matches of increasing frequency, or under varied conditions. Participants completed this task online, on a personal computer. Play-time was limited to 20 min for all conditions, after which a pop-up appeared automatically and transferred participants to the next task.

#### In-game rewards

Reward presentation and availability varied across conditions. Three rewards—each with different gameplay functions—were available. Rewards were all in-game power-ups common to match-3 games, allowing players to destroy a single candy of their choice, to switch the position of two candies, or to destroy all candies of a randomly determined colour. These power-ups helped players complete level objectives, but varied in the level of support they afforded. Participants were provided with instructions indicating the functions and hierarchy of the power-ups. These rewards were modelled after commonly available rewards in this genre of game, enhancing the ecological validity of our experimental task.

Rewards were available only in the Fixed and Loot Box conditions (see supplemental materials, SI Fig. 2), with the opportunity to purchase rewards presented to participants at the end of each level. In the Fixed Reward condition, rewards were presented in a marketplace-style buying window. In the Loot Box condition, a single option was presented to participants to purchase a loot box containing a random item from the 3-item pool of rewards. Thus, unlike the Fixed Reward condition, in the Loot Box condition the outcome of the reward purchase was unknown to players prior to purchase. In the No Reward (control) condition, rewards were unavailable, and their access was hidden. The gameplay itself, however, was unchanged. Importantly, because the same rewards were available in both the Fixed-reward and Loot Box conditions, the value of available rewards did not systematically vary by condition. This ensured the robustness of our experimental manipulation, ensuring that any differences observed between conditions were due to the randomisation of the reward delivery and not the value of the rewards themselves.

Rewards were purchased using virtual currency earned through gameplay and items were equally valued across the Fixed Reward and Loot Box conditions (200 coins). Once purchased, rewards were available to use during gameplay at the players’ discretion.

### Measures

#### Balloon analogue risk task (BART)

The BART^59^ is a computerised task designed to measure individual risk-taking *behaviour*. Instructions for the BART were presented on-screen at the commencement of the task. In our study, 30 balloons (trials) were presented to each participant. Each trial required participants to simulate the inflation of a balloon, knowing that an undetermined number of inflations would pop the balloon. The value for the pop threshold varied between 2 and 14 inflations and was randomised for each trial. With each inflation, participants accumulated tokens, and if the balloon popped the tokens were lost. Thus, the risk of loss accumulates with each additional inflation of the balloon. After each inflation, participants can either cash-out their tokens or inflate again. Choosing to cash out transferred the tokens accumulated for that trial into a bank, ending the current trial, and commencing the next. Banked tokens are safe and cannot be lost in subsequent trials. Importantly, this task is designed such that risk is correlated with reward^62^. Performance on this task was measured as the adjusted average number of pumps on unexploded balloons (i.e., the average across participants, of pumps where the balloon did not explode), with higher scores indicative of greater risk-taking behaviour^59,60^. To add value to the tokens accumulated in this task, every token retained at the end of the task bought participants an additional entry into a prize draw for one of six $50 gift vouchers.

#### The problem gambling severity index (PGSI)

The PGSI^63^ is a nine-item survey asking the frequency with which participants engage in a variety of gambling-related activities over the past 12 months (e.g., “Have you needed to gamble with larger amounts of money to get the same feeling of excitement?”, “Have you borrowed money or sold anything to get money to gamble?”). Recent validation studies of the PGSI have recommended collapsing the gambling categories from four groups into three, by rescoring the groups as *low-risk* (1—4), *moderate-risk* (5—7), and *problem gambler* (> 7) as recommended by Currie et al.^64^. This recalibrated variation is commonly used in loot box research (e.g., ^15,53^ and was employed in this study. Internal reliability for this measure is high (α = 0.936).

#### The risky loot-box index (RLI)

The RLI is a five-item scale designed to examine risky engagement with loot boxes^38^. Participants are asked to indicate the extent of their agreement with statements like: "The thrill of opening loot boxes has encouraged me to buy more" and "I have put off other activities, work, or chores to be able to earn or buy more loot boxes" on a Likert scale ranging from 1 (*strongly disagree*) – 7 (*strongly agree*). Internal reliability for this measure is high (α = 0.915).

### Procedure

Participants were alternatingly allocated to conditions in temporal order of recruitment (i.e., first: control, second: loot box condition, third: control, etc.). After allocation, participants joined an online video-conferencing session with the researcher. Participants were guided in the registration process needed to access the online data collection platform for the experiment and provided with gameplay and reward information relevant to their condition. Participants then played the game freely for the allotted 20 min, before proceeding with the BART. At the completion of the two tasks, participants completed demographic questions, followed by the RLI, the PGSI, and some additional items unrelated to the present study. At the conclusion of these items, participants were thanked for their time and debriefed.

### Statistical analysis

Data cleaning and consolidation was conducted in Microsoft Excel. Analysis and transformations were conducted in Jamovi^65^. Bayesian t-tests were conducted using the *jsq* add-on, and moderation analysis through the *medmod* add-on. A Bayesian approach to analysis offers several advantages over the more common NHST, frequentist approach. Although a full consideration of these advantages is beyond the scope of the present paper, an excellent treatment of this topic can be found in Wagenmakers, Marsman et al.^66^. Of primary importance here, however, is that Bayesian analyses allow us to quantify evidence in favour of a null effect. Thus, if our manipulation fails to produce an effect on behavioural risk-taking, we can say something meaningful about the strength of evidence supporting this null effect (i.e., that levels of behavioural risk-taking are similar across conditions); rather than simply concluding that we failed to find evidence *for* an effect. Bayes factors are interpreted in line with Wagenmakers, Love et al.^67^. A Bayes factor of 1–3 is considered anecdotal evidence, 3–10 is moderate evidence, 10–30 is strong, 30–100 is very strong and > 100 is extreme evidence for the alternative (or null) hypothesis. Effect size is indexed by δ, the default measure provided by the JAMOVI software. δ is the population equivalent of Cohen’s *d* (effectively the standardised mean difference between groups), with cut-offs of 0.2., 0.5., and 0.8 for small, medium, and large effects, respectively.

## Results

Data are available at: https://osf.io/tqcmg/.

### The effects of reward condition on risk-taking

First, we established that players engaged with the reward mechanisms. Players varied in the extent to which they engaged with the reward mechanisms, but there was evidence of engagement in both reward conditions. On average, players in the Loot Box condition purchased 12 rewards (range 0–46) and used four rewards (range: 0–18), and players in the Fixed Rewards conditions purchased 12 rewards (range 0–42) and used four rewards (range: 0–33).

Next, we ran Bayesian, one-sided, independent samples t-tests compared mean BART scores between groups. Our a priori hypotheses for H1 & H2 specified a directional prior distribution for an alternative hypothesis in which Group 1 > Group 2 (Loot Box > Other Groups). Given the exploratory nature of these reward group comparisons, we did not have justification to deviate from the default Cauchy prior distribution (0.707)^67^.

**Figure 1:**
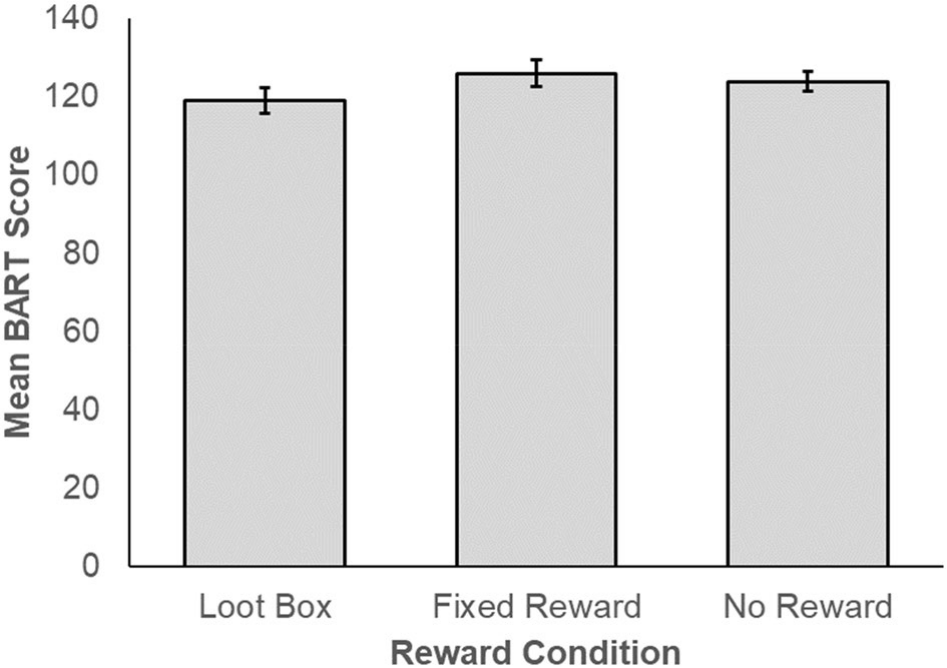
Mean BART scores according to reward condition.

**Figure 2:**
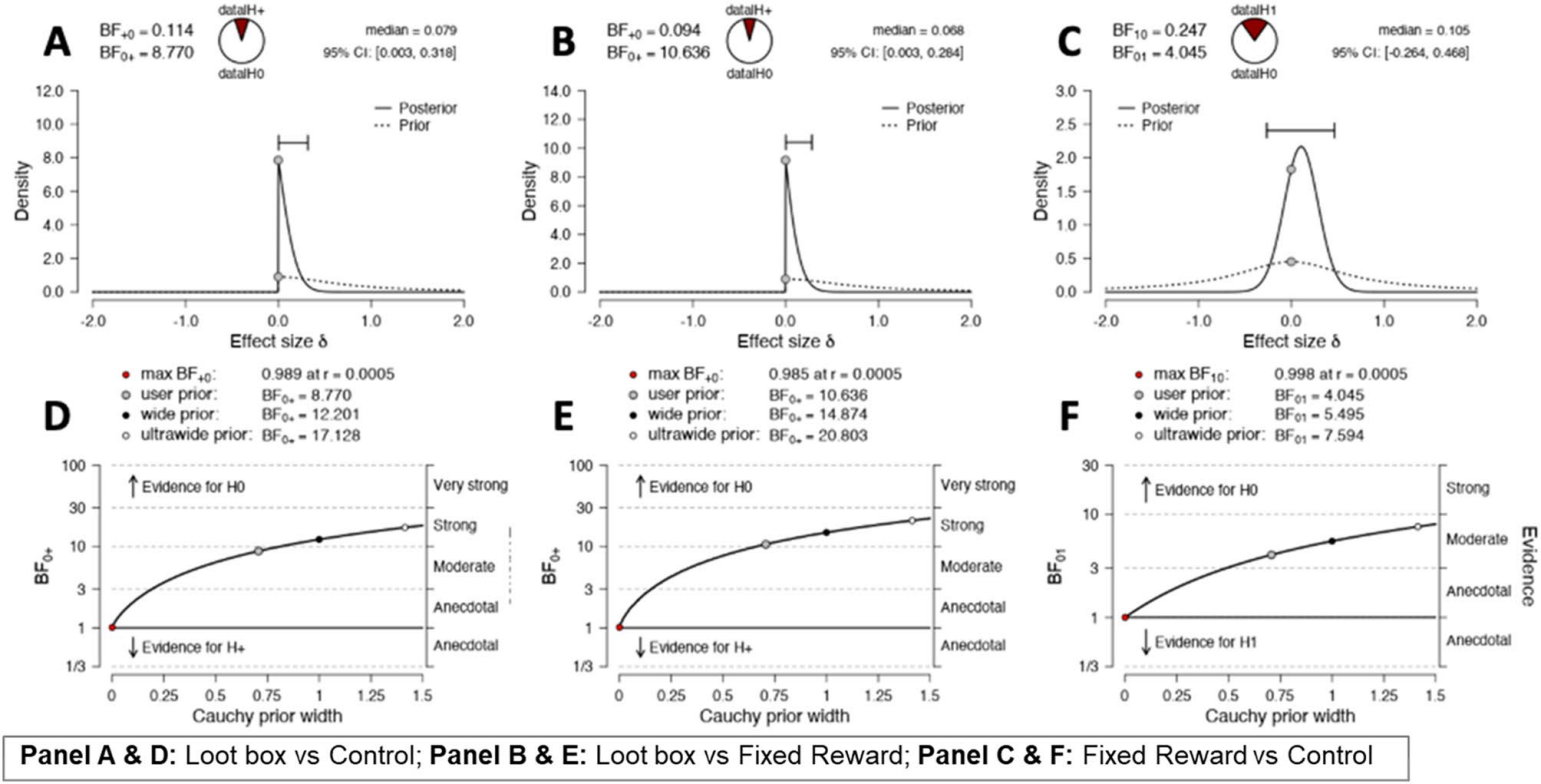
Prior and Posterior Plots, and Robustness Checks for Bayesian T-Tests.

Our analyses returned three key, simple findings regarding the relationship between reward condition and risk-taking. First, mean risk-taking scores on the BART were very similar across reward conditions (Fig. 1). When comparing risk-taking in the Loot box and Control conditions (H1), a Bayesian independent samples t-test returned *moderate* evidence in favour of the null hypothesis (BF_0+_  = 8.77, indicating that the data are 8.77 times more likely under the null than the alternative hypothesis; Fig. 2A). When comparing the Loot Box and Fixed Reward Groups (H2), we found *strong* evidence in favour of the null hypothesis was observed between Loot Box and Fixed Reward Groups (BF_0+_  = 10.64; Fig. 2B). Finally, an exploratory two-sided, independent samples Bayesian t-test compared BART scores between the Fixed Reward and No Reward groups (H3). Again, we found *moderate* evidence (BF_01_ = 4.05) in favour of the null hypothesis (Fig. 2C). Consistent with the observed evidence for null effects, median effect sizes were very small (δ = 0.07–0.11; see *median* values in Fig. 2A–C). As can be seen in Fig. 2, the observed median effect sizes fall below or, in one case, close to the cut-off for a small effect (δ = 0.1).

Second, robustness checks for our t-tests—indicating the extent to which the posterior odds vary as a function of the selected prior—suggest that the data provide *moderate* to *strong* evidence in favour of the null hypotheses across a wide range of plausible prior distributions (Fig. 2D–F). Thus, the obtained results are not dependent on our use of the default prior.

**Figure 3:**
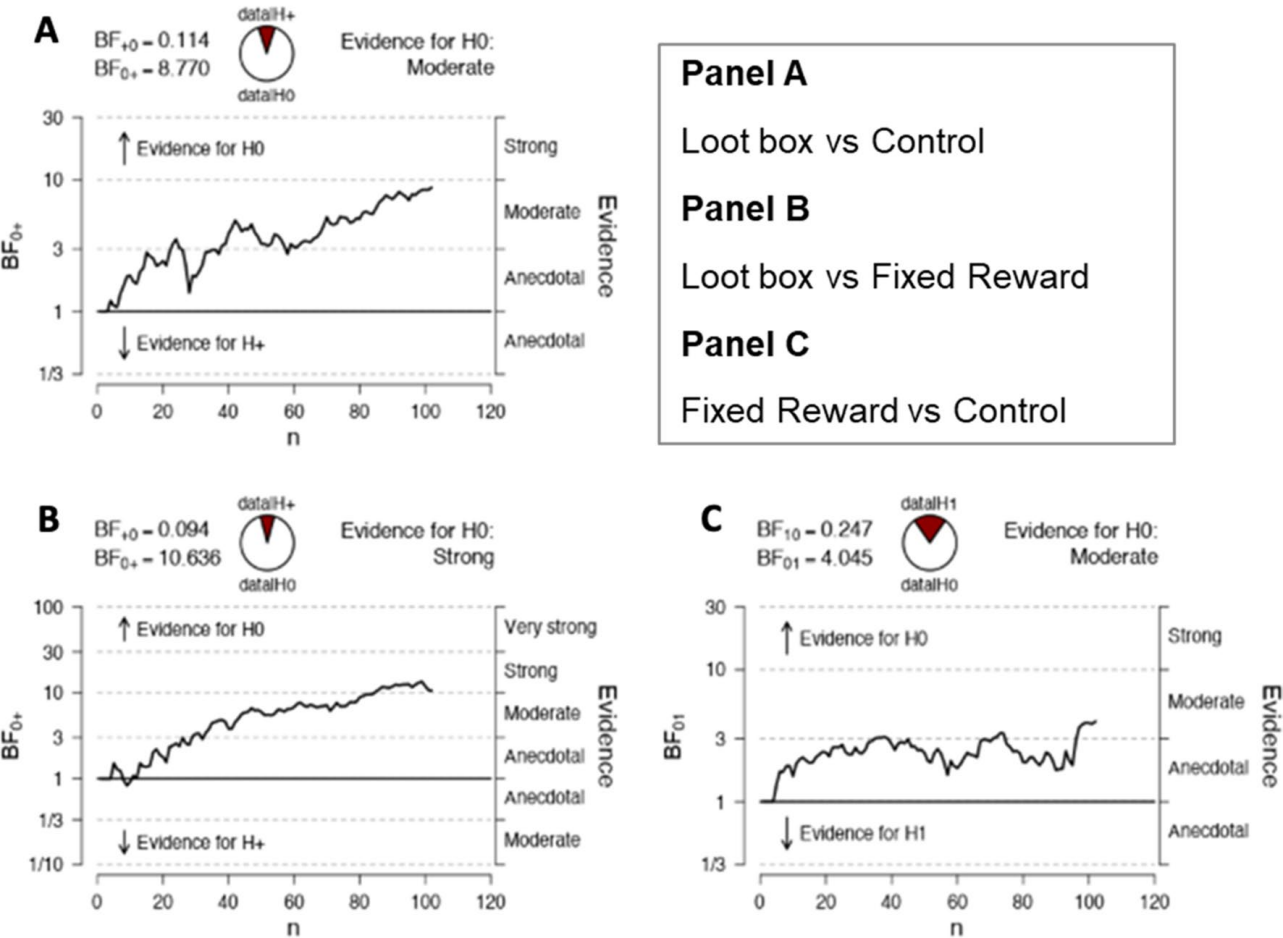
Sequential analyses for Bayesian T-tests.

Finally, sequential analyses show that, for H1 and H2 comparing BART scores for the randomised condition to those for the no reward and fixed reward conditions, as data accumulated the evidence in favour of the null hypotheses strengthened in a generally systematic manner (Fig. 3A and B). For H3, comparing BART scores for the fixed and no reward conditions, the strength of evidence for the null plateaued and hovered around the cut-off for moderate strength (Fig. 3C).

Combined, these results suggest that our data provide compelling evidence against an effect of exposure to gambling-like in-game rewards on subsequent risk-taking; at least in the present study.

### Moderation analyses

The literature suggests that previous gambling history may influence individual risk-taking. Thus, we tested whether PGSI score moderated the relationship between exposure to gambling-like rewards and BART scores. We hypothesised that differences in BART scores between the Randomised reward condition and two controls would be greater for participants with higher scores on the PGSI (H4). Given the absence of accessible Bayesian tools for moderation, we reverted to a Frequentist, null hypothesis significant testing approach. Estimate robustness was increased by bootstrapping to 1000 samples.

**Figure 4:**
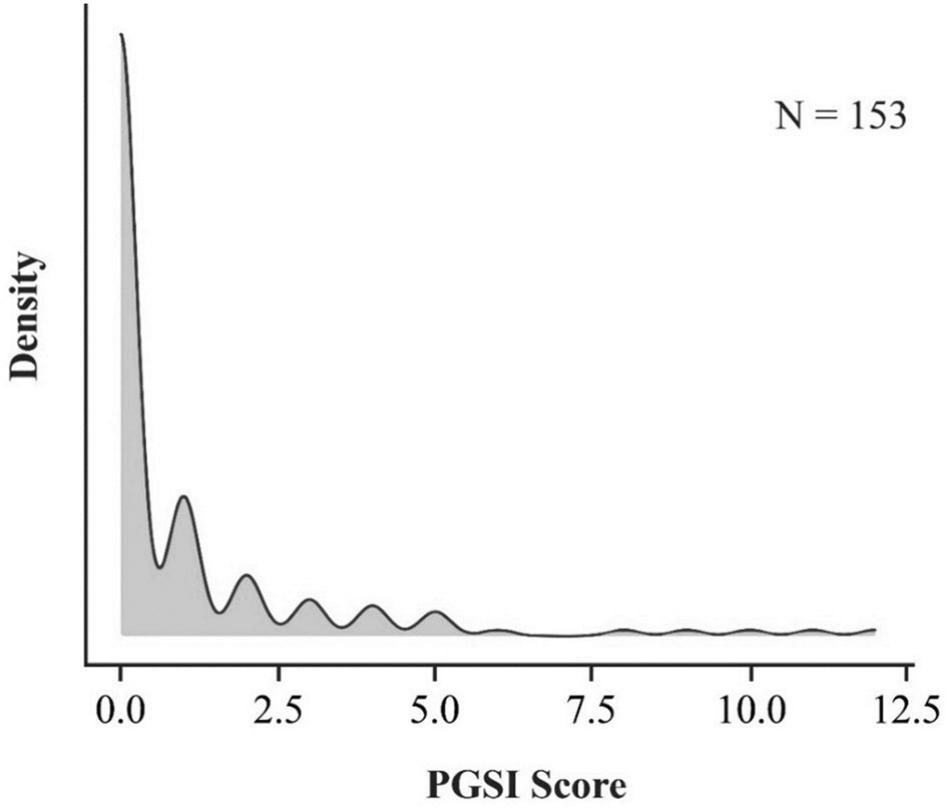
Density plot of PGSI score distribution.

We found no evidence that PGSI moderated the effect of reward condition on BART scores when comparing the Randomised Reward condition to either the No Reward control (H4) or the Fixed Reward condition (H5), b = 0.32, 95% CI [− 3.40, 3.50], *p* = 0.854 and b = 2.11, 95% CI [− 4.0, 8.85], *p* = 0.530, respectively. This may, in part, reflect the distribution of PGSI scores in our sample, which was positively skewed, indicating a small proportion of gambling activity (Fig. 4). Specifically, in our sample, 70% of participants (N = 99) were categorised as non-gamblers, 29% (N = 44) were categorized as low risk gamblers (PGSI 1–4). Only 5 participants were categorized as moderate risk gamblers (PGSI 5–7), and only 2 were categorised as high-risk gamblers (PGSI 7+).

### Correlations

We also explored whether risk-taking was correlated with risky loot box use, as measured by the RLI (H6). Further, and as a replication of previous research^38^, we explored correlations between the PGSI and RLI (H7). Interestingly, a Bayesian correlation showed moderate evidence against a relationship between BART and RLI scores (*r* = − 0.06, BF_01_ = 7.72). However, we conceptually replicated Brooks and Clark’s^38^ significant, positive correlation between the PGSI and RLI, finding very strong evidence for a positive correlation using a Bayesian approach (*r* = 0.35, BF_10_ = 2197 when Spearman’s rho was used to counter the skew in the PGSI data).

## Discussion

Loot boxes are psychologically akin to conventional gambling activities^6^, and increased loot box engagement is positively associated with problem gambling severity^16^. These theoretical and empirical associations between loot boxes and gambling have prompted speculation that loot box engagement may lead to future gambling behaviour. Simulated gambling activities can affect risk assessment, thus an effect on subsequent risk-taking is one mechanistic pathway through which loot boxes might encourage future gambling. We experimentally tested the behavioural effects of engaging with loot boxes, compared to fixed and no reward gameplay conditions, on subsequent risk-taking behaviour. We found no meaningful differences between conditions in player’s subsequent risk-taking behaviour as measured by the BART. In fact, we found moderate-to-strong evidence against such an effect. To the extent that the BART is a valid measure of risk-taking behaviour, and our experimental task is ecologically valid, the findings suggest that risk-taking behaviour is not increased by exposure to loot boxes. We discuss the limitations and boundary conditions of our findings below. The observed null effect was also not moderated by problem gambling symptomatology though, again, we must interpret this absence of moderation cautiously given the low number of participants in our sample with moderate risk and problem gambling symptomatology.

These findings have multiple interpretations. If taken on face value, our findings may indicate that loot box engagement does not affect subsequent risk-taking, and speak against this pathway for a “gateway” hypothesis. Three aspects of our research, in particular, enhance our confidence in the utility of our findings. First, we pre-registered our design, hypotheses, and analyses, reducing researcher degrees of freedom and thereby limiting researcher-driven Type-1 error. Second, rather than relying on self-reported measures of loot box engagement and risky behaviour, we experimentally manipulated exposure to, and facilitated engagement with, loot boxes and measured subsequent behavioural risk-taking. We created an engaging and novel experimental task (our video game), which allowed us to design, manipulate, and implement our reward systems in line with the key psychological constructs of interest, and to directly measure our behavioural outcome of interest. The limitations in attempting to simulate the real-world gaming experience of loot box engagement in our experiment are discussed below. However, participant feedback on the game task was positive (i.e., the game was genuinely enjoyable), and encouraging for future work. Finally, using a Bayesian approach to analysis allowed us to quantify the evidence against an effect, rather than simply conclude that we were unable to find evidence *for* an effect. Thus, our results speak to the evidence *against* this mechanistic pathway, not simply the absence of evidence *for* this pathway.

Despite these strengths, aspects of our design may also have constrained the relationship between our manipulation of reward-type and risk. First, gameplay might not have been long enough, and/or the in-game rewards may not have been perceived to be valuable enough, for participants to reach the level of engagement required to generate effects. Risk-taking is tied to perceptions of value for both what is being wagered and what might be won. When considering in-game rewards, value can be derived from the importance placed on in-game items in the gaming context (i.e., apart from any value associated with the ability to on-sell items). However, such value will likely be moderated by the extent to which the gamer is engaged with the game and the rewards; the more engaged a gamer is, the more subjective value in-game items are likely to have. Thus, the short playtime in our experiment and the fact that rewards were only useful within the context of the game may have constrained player engagement, and the value placed on in-game rewards. This, in turn, may have limited the sense of risk involved with loot box engagement, and subsequent effects on risk-taking behaviour. We acknowledge that longer gameplay times, deeper game investment or engagement, and more valuable rewards may produce the hypothesised effect, though further research is required to explicitly test these possibilities. Second, and on a related note, the virtual currency with which rewards were purchased might have been too easily earned, reducing its perceived value and, again, the extent to which engagement with loot boxes was perceived as “risky” behaviour (i.e., the currency lack value, there is little risk in exchanging it for an unknown outcome). We anticipated that some participants might score within the *problem gambler* range (> 7) on the PGSI^68^. To mitigate risks to such participants (i.e., posed by exposure to gambling-like reward mechanisms), we used virtual currency instead of real money for reward purchases. Our efforts to balance our desire for ecological validity with a concern for participant safety may have undermined the perceived value of in-game rewards, and subsequent risk and reinforcement effects. Finally, the BART may not be the best measure of risk in this context. The construct of risk is undergoing conceptual revision, with increased attention paid to measuring self-reported risk propensity, risk frequency, and actual risk behaviour^58^. Although the BART (and other behavioural tasks) speak to risk behaviour, they have been shown to correlate poorly with risk propensity and frequency measures^58^. Thus, the BART may have been relatively insensitive to the cognitive processes underlying and influencing loot box and reward engagement in our experiment. However, our primary interest was in effects on risk-taking behaviour. Thus, we thought the BART suitable for this initial study because (a) it offers a behavioural measure of risk-taking, (b) it has been validated^59,60^ and used widely in the literature on risk-taking^58,62,69^, and (c) we were able to gamify the task, which allowed it to fit neatly into our paradigm and maintain the focus on risk-taking in a video game context. Nonetheless, we acknowledge that conceptual replication using other measures of risk-taking is required to ensure that our null effects are not due to idiosyncrasies of the BART. For empirical examination of differences in risk preferences and risk-taking, see Pedroni et al.^70^

### Risk-taking behaviour, risky loot box use, and problem gambling symptomatology

In addition to the effects of engaging with randomised rewards on subsequent risk-taking behaviour, our data addressed two other issues of interest. First, we sought to correlate our measure of behavioural risk-taking (the BART) with self-reported measure of loot-box related risk-taking (the RLI;^38^. We found moderate evidence against a meaningful correlation between risk-taking behaviour as measured by the BART and self-reported risky loot box use. Despite its name, the Risky Loot-Box Index includes questions related, but not limited to, risk-taking (as adapted from the financial subscale of the DOSPERT;^71^). In contrast, the BART is a more generalised risk-taking behavioural measure, and may not adequately capture the same risk-related influence of loot box engagement as the RLI. Further, the RLI could be considered a self-report measure of risk *propensity* related specifically to loot box engagement. As mentioned, behavioural measures of risk-taking can correlate poorly with measures of risk-taking propensity^58^. The poor correlation observed between the BART and RLI may reflect differences in the domain of risk-taking measured (i.e., propensity vs behaviour), or important context-specific differences (e.g., between generalised risk-taking behaviour and loot box-specific risky behaviour). Alternatively, the limited variation in RLI scores and previous loot box engagement within our sample may have constrained the correlation between measures.

Finally, we replicated previous correlations reported between PGSI and RLI^38^. This recurrent correlation underlies the rationale for the present research: that self-reported risky loot box spending co-occurs with problem gambling symptomatology. This reinforces concerns that spending on loot boxes might be in part driven by vulnerable gamers with problem gambling symptoms.

### Future directions

Following the identified limitations of the current research, we recommend three directions for further investigation. First, the allotted gameplay (and subsequent reward exposure) *time* in our experiment may have constrained participants’ perceptions of reward value or purchase risk. Future work using experimental manipulations of gameplay conditions and reward exposure may benefit from increasing the perceived value of rewards (i.e., making them more essential to game progression) and/or increasing the perceived value of the in-game currency used to purchase these rewards. This may aid in creating a genuine sense of risk, loss, and risk-reward ratio in purchasing loot boxes.

Second, the single exposure-phase in this experimental design may have constrained player engagement. In reality, players’ experience and engage with loot boxes over multiple gaming sessions and prolonged time periods^18,53^. This repeated exposure to the reward mechanisms, and investment of time in the associated game, may change perceptions of reward value and associated purchasing risk. Future work may benefit from a longitudinal design, encouraging multiple gameplay session over a longer period, and allowing players more time to become invested in the game and to value the rewards in the context of that investment. Designs with extended gameplay would also allow for in-game currency to be more difficult/timely to accumulate and, therefore, more valuable (further enhancing perceptions of risk associated with reward purchases).

Finally, conceptual replication is required to ensure our null findings are robust to variations in measurement of risk-taking; particularly considering recent developments in risk conceptualisation^62,69^. Alternative behavioural measures (e.g., the Iowa Gambling Task), and measures of risk preference, may identify cognitive and behavioural effects of loot box engagement undetected in the current work.

## Conclusion

Consumers, academics, and policy makers have expressed concerns that loot boxes may be adversely influencing gamers through predatory design and implementation. Some have speculated that loot boxes engage cognitive processes that may promote future gambling behaviour. Our research is the first to directly test one pathway—effects on risk-taking—through which engagement with loot boxes might encourage future gambling. We found evidence against this pathway: Engaging with loot boxes did not increase subsequent risk-taking behaviour. However, we place two essential caveats on this conclusion. First, ours is only the first study to directly test the relationship between loot box engagement and risk-taking behaviour. Second, gaming behaviour—and spending on in-game rewards—operates in a complex system of personal and social motivations. Limitations inherent in any effort to translate complex, real-world behaviour to controlled experimental settings demand caution when generalising findings. Nonetheless, our results appear to speak against the presence of a gaming-gambling gateway effect, and add support to the notion that problem gambling symptomatology predicts loot box spending rather than being caused by it.

## Supplementary Information

Supplementary Information.

## Data Availability

Data are available at: https://osf.io/tqcmg/.
