# Supplementary material for: No effect of short term exposure to gambling like reward systems on post game risk taking
Source: Sci Rep. 2022 Oct 6;12:16751. doi: 10.1038/s41598-022-21222-3 (PMC9537418; doi:10.1038/s41598-022-21222-3)
Supplement: Supplementary file 1 — Supplementary Information. [file 41598_2022_21222_MOESM1_ESM.docx]

**Supplemental Materials**

**SI Figure 1**

Experimental Video Game Software**
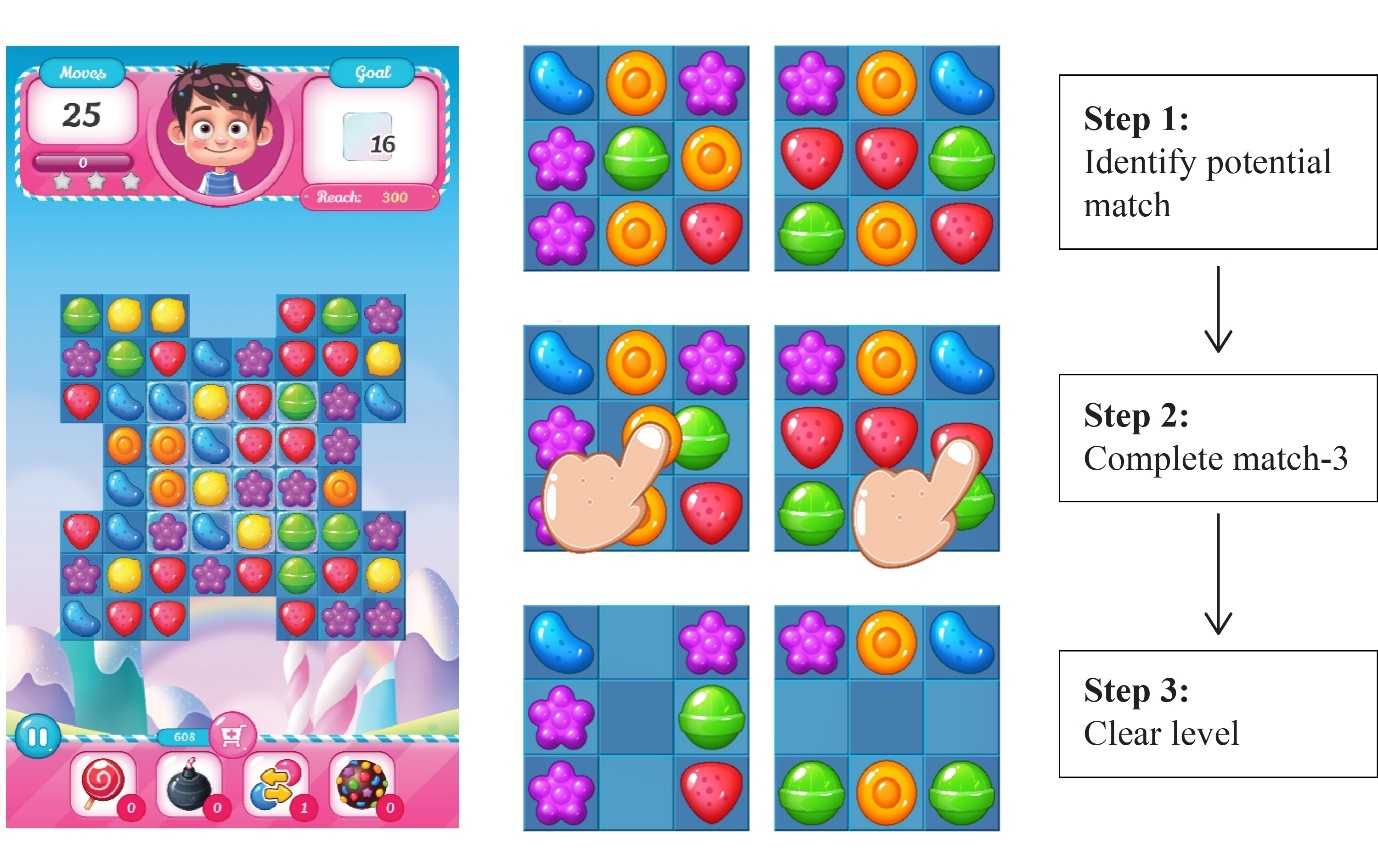
**

**Credit: Screenshot of game developed by Dr. Lewis in Unity using assets purchased from the Unity store under an EULA agreement from gamevanilla (gamevanilla.com).**

**SI Figure 2**

Reward Interface per Condition


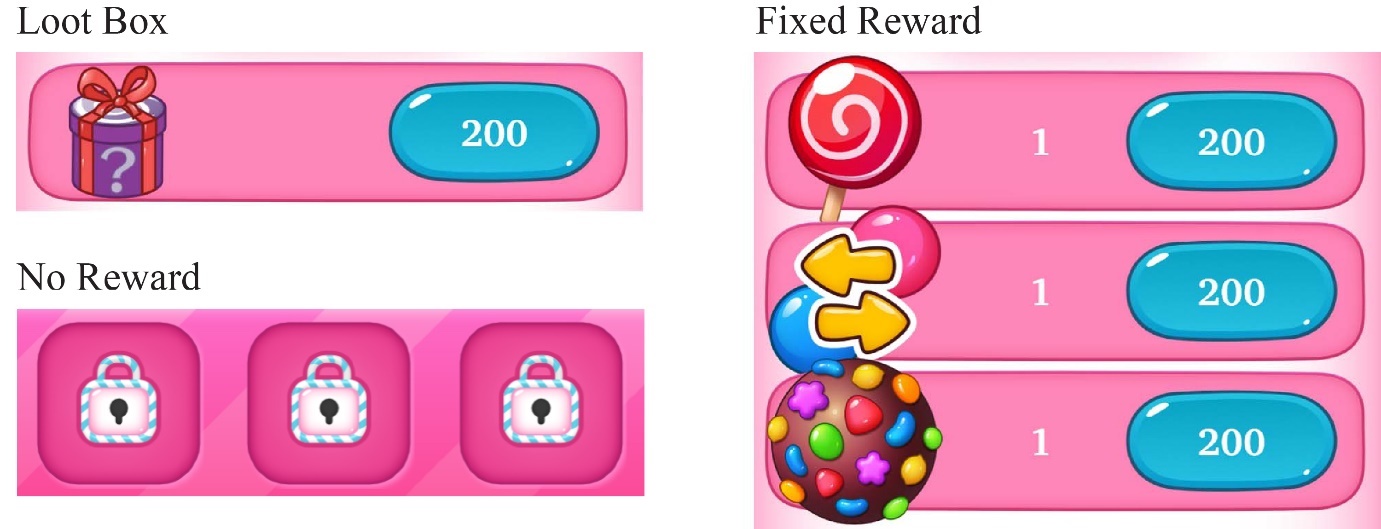


**Credit: Screenshot of game developed by Dr. Lewis in Unity using assets purchased from the Unity store under an EULA agreement from gamevanilla (gamevanilla.com)**

**Analyses including the data collected in 2020**

As noted in the main text, data collection for this study began in 2020 but was interrupted by the COVID19 pandemic, and only 26 participants were tested. We began data collection anew, and collected a full sample of 153 participants, in 2021. For transparency we report the analyses from the main text including the additional data collected in the aborted project in 2020. Including the data collected during 2020 adds a further 26 participants to the sample.

The Effects of Reward Condition on Risk-Taking.

Bayesian, one-sided, independent samples t-tests compared mean BART scores between groups. A priori hypotheses for H1 & H2 specified a directional prior distribution for an alternative hypothesis in which Group 1 > Group 2 (Loot Box > Other Groups). Given the exploratory nature of these reward group comparisons, we did not have justification to deviate from the default Cauchy prior distribution (0.707) (Wagenmakers, Love, et al., 2018). Table S1 shows the mean (SD) BART scores for the three conditions. We provide the corresponding statistics for the final sample (i.e., reported in the manuscript) for ease of reference. Table S2 reports the Bayes factors and effect sizes for the Bayesian independent samples t-tests testing H1, H2, and H3. As can be seen, including the initial 26 participants’ data did not materially affect conclusions.

**SI Table 1. Means (SD) BART Scores According to Condition for Full and Final Samples.**

| Reward Condition | Full Sample (N = 179) | Final Sample (N = 153) |
| --- | --- | --- |
| Loot Box | 117 (24.6) | 119 (23.6) |
| Fixed Reward | 122 (26.3) | 126 (24.3) |
| Control | 118 (22.0) | 124 (17.7) |

**SI Table 2. Bayes Factors and Effects Sizes for Tests of H1, H2, and H3.**

| Hypothesis | Full Sample (N = 179) | Final Sample (N = 153) |
| --- | --- | --- |
| H1 (Loot vs Control) | BF_0+_ = 6.402; δ = 0.10 | BF_0+_ = 8.770; δ = 0.08 |
| H2 (Loot vs Fixed) | BF_0+_ = 10.274; δ = 0.07 | BF_0+_ = 10.636; δ = 0.07 |
| H3 (Fixed vs Control) | BF_01_ = 3.485; δ = 0.15 | BF_01_ = 4.045; δ = 0.11 |

**Moderation Analyses**

We tested whether PGSI moderated the effect of reward condition on BART scores when comparing the Loot Box condition to either the No Reward control (H4) or the Fixed Reward condition (H5). The coefficients, 95% CIs and *p* values for the relevant interaction term (Reward Condition * PGSI) are provided in Table S3, together with the corresponding statistics as reported in the manuscript. Again, the conclusions do not change when the data from the initial 26 participants are included.

**SI Table 3. Moderation Analyses Testing H4 and H5.**

|  | Full Sample (N = 179) | | | Final Sample (N = 153) | | |
| --- | --- | --- | --- | --- | --- | --- |
| Hypothesis | *b* | 95% CI | *p* | *b* | 95% CI | *p* |
| H4 (Loot vs Control) | .92 | -3.10, 4.32 | .624 | 0.32 | -3.40, 3.50 | .854 |
| H5 (Loot vs Fixed) | -2.22 | -5.22, 7.41 | .530 | 2.11 | -4.0, 8.85 | .530 |

**Correlations**

We used Bayesian correlations to test for evidence of associations between BART and RLI (H6), and PGSI and RLI (H7). The correlations coefficients and Bayes factors for these tests based on the full sample are presented in Table S4, alongside the correlation based on the final sample (and reported in the manuscript). Again, the inclusion of the initial 26 participants’ data did not materially affect conclusions.

**SI Table 4. Bayes Factors and Correlations Coefficients for Tests of H6 and H7.**

| Hypothesis | Full Sample (N = 179) | Final Sample (N = 153) |
| --- | --- | --- |
| H6 (BART*RLI) | BF_01_ = 6.14; δ = -.08 | BF_01_ = 7.72; δ = -.06 |
| H7 (PGSI*RLI) | BF_10_ = 3163; *r* = .34 | BF_10_ = 2197; *r* = .35 |
